# Predisposing Factors to Premature Coronary Artery Disease in Young (Age ≤ 45 Years) Smokers: A Single Center Retrospective Case Control Study from India

**DOI:** 10.5681/jcvtr.2014.003

**Published:** 2014-03-21

**Authors:** Amitesh Aggarwal, Sourabh Aggarwal, Prattaya Guha Sarkar, Vishal Sharma

**Affiliations:** Department of Medicine, University College of Medical Sciences (University of Delhi) and GTB Hospital, Delhi, India

**Keywords:** Coronary Artery Disease, Young, Smoking, Dyslipidemia, Diabetes, Hypertension, Obesity

## Abstract

***Introduction:*** The role of the conventional risk factors in premature coronary artery disease (CAD) after eliminating the confounding variability of smoking has not been evaluated. This study was conducted to identify role of traditional risk factors in smokers with premature CAD.

***Methods:*** The case records of patients presenting acutely with premature CAD during the period 2007-2010 were analyzed retrospectively. Age, sex and smoking matched controls were selected from same time period. Data records were obtained for family history, alcohol, waist size, blood pressure, hypertension, blood sugar, lipid profile and presence of cutaneous markers for both groups and analyzed using statistical software.

***Results:*** 234 smokers with CAD and 122 smokers without CAD were included in groups 1 and 2, respectively. The patients in group 1 had significantly increased prevalence of hypertension, diabetes mellitus, metabolic syndrome, dyslipidemia and central obesity. There was no difference in prevalence of family history of CAD, arcus juvenilis and baldness. We found statistically significant association of hypertension, DM and metabolic syndrome in young smokers with premature acute CAD in Indian population as compared to young smokers without CAD.

***Conclusion:*** In young smokers, presence of hypertension, central obesity, diabetes mellitus and metabolic syndrome identifies a subset at increased risk for future acute CAD requiring more rigorous follow up and treatment.

## Introduction

Coronary artery disease (CAD) is a leading cause of morbidity and mortality in both developing and developed countries. CAD is also the most common cause of mortality in India, where approximately one-sixth of the world population lives.^1^ It is a well-established fact that the South Asian population, especially Indian sub-continent, has higher risk and wider prevalence of CAD as compared to rest of ethnic groups.^2,3^ CAD has been reported to manifest itself in the subcontinent almost a decade earlier than that in the west.^4,5^ Moreover, deaths related to CAD have been found to occur 5 to 10 years earlier in Indian sub-continent than in Western countries.^6^

Smoking has been shown to be one of the strongest risk factor for patients with premature CAD.^7-9^ The association of a positive family history of CAD, dyslipidemia, hypertension, diabetes mellitus (DM), central obesity and metabolic syndrome (MS) with CAD is known to us and recent studies have also shown the association of these risk factors with premature CAD too.^10-14^ However, in all of the performed studies, smoking has been a potential confounding factor since its independent association with other risk factors is a known fact. There is a paucity of data on studies evaluating the role of these conventional risk factors especially in the premature CAD patients (≤40 years) after eliminating the confounding variability of smoking. Thus, this retrospective case-control study was planned to elucidate the factors predisposing young smokers (≤40 years) to premature CAD and establish their association with premature CAD independent of smoking.

## Materials and methods

The present retrospective case control study was conducted at a 1600 bedded tertiary care teaching hospital in New Delhi. The case records of all the patients presenting acutely with premature CAD (age ≤40 years) during the period 2007-2010 were analyzed retrospectively. Records of only those subjects who presented with acute myocardial infarction or acute coronary syndrome, and were hospitalized to the coronary care unit (CCU) of the hospital were included for analysis. The diagnosis of acute coronary syndrome was made on the basis of symptoms, electrocardiogram (ECG) abnormalities, cardiac injury enzymes and/or echocardiography as described by Luepker et al.^15^ The case sheets were scrutinized for smoking status with intensities, lipid profile, DM, hypertension and family history of CAD. Records were screened for presence of cutaneous markers viz., arcus juvenilis, premature graying of hair and premature balding. Incomplete case records with missing data, subjects who had presented as brought dead to the hospital or those who had unexplained deaths were excluded from the study.

In order to obtain adequate control population, records were searched for age, gender and smoking index matched patients who presented to our smoking cessation clinic with non-cardiac complaints during same time period. All patients with previous history of CAD and those who were admitted during their acute presentation were excluded to remove elements of confounding bias. Only those patients were selected for whom the complete data were available. After reviewing all the available data very carefully, 122 patients were selected for controls.

Particular note as made of waist size, fasting and post prandial blood sugar, fasting lipid profile (total cholesterol, high density lipoprotein cholesterol (HDL-C), low density lipoprotein cholesterol (LDL-C), triglycerides) and measurement of carotid intima media thickness (CIMT). Smoking was defined as use of bidi (bidis are small hand-rolled cigarettes wrapped in a piece of local tobacco leaf), cigarette or oral tobacco. Consumption of 1 gram of oral tobacco was taken as equivalent of smoking of one cigarette. The grading of smoking was assessed by calculating smoking index (SI), which was based on average number of cigarettes smoked per day multiplied by total duration in years of active smoking. Dyslipidemia was considered as per NCEP-ATP III guidelines.^16^ Hypertension was diagnosed according to JNC 7 criteria.^17^ Diabetes mellitus was defined as per American Diabetes Association, 2004 criteria.^18^ Prediabetes was defined as fasting blood sugar between 100-125 mg/dl impaired fasting glucose (IFG) and post prandial blood sugar between 140-199 mg/ dl impaired glucose tolerance (IGT). A 25% or more graying of scalp and /or beard hair on visual inspection was taken as presence of premature graying in records.^19^ Balding had been defined using Norwood-Hamilton scale.^20^

For the purpose of study, the criterion advocated by International Diabetes Federation (IDF) consensus 2005, which lays impetus on ethnic inheritance in diagnosis of obesity, was used for diagnosing patients with MS.^21^ Thus, any patient presenting with abdominal obesity (defined by waist circumference ≥90 cm in males and ≥80 cm in females) plus two or more of the following risk factors was diagnosed with metabolic syndrome:

1. Blood pressure ≥130/≥85 mmHg or treatment of previously diagnosed hypertension
2. Fasting blood glucose ≥100 mg/dL or previously diagnosed type 2 diabetes
3. Triglyceride ≥150 mg/dL or specific treatment for this lipid abnormality
4. HDL cholesterol <40 mg/dL in males and <50 mg/dL in females or specific treatment for this lipid abnormality

### Statistical analysis

Statistical analysis was performed using SPSS 17 (SPSS Inc., Chicago, IL, USA). Continuous variables were expressed as mean± standard deviation (SD) and percentage was calculated for categorical variables. Median and inter-quartile range was calculated for variables with non-normal distribution. Comparison of continuous and categorical variables was done in male and female group using unpaired 2-tail t-test and Fisher’s exact test and any statistically significant difference was noted. Odds ratio (OR) was calculated for risk factors with discrete variables. For the purpose of the study, p≤ 0.05 was considered statistically significant.

## Results

Medical records of a total of 310 young patients (≤40 years) with acute CAD were initially studied and after applying inclusion and exclusion criteria, 234 patients were selected for study and included in group 1. Out of 234 patients, 226 (96.58%) were males and 8 (3.42%) were females. Mean age at the time of admission was 35.45 ± 0.25years. In control group (group 2), 184 patients’ data were studied after inclusion and exclusion criteria, 122 patients were selected with 116 (95.08%) being males and 6 (4.92%) being females. Mean age of patients in group 2 was 34.35 ± 0.62 years. Various metabolic parameters of smokers with CAD (group 1) and smokers without CAD (group 2) are compared in Table 1.

**Table 1:** Comparison of risk parameters between two groups

	**Group 1 (n=234)**	**Group 2 (n=122)**	*P*	**Odds ratio**
Age in years(mean ± SD)	35.45 ± 0.25	34.35 ± 0.62	0.10	-
Males	226 (96.58%)	116 (95.08%)	0.56	-
Smoking index	Median 157 Range 1498 Inter quartile Range 225	Median 120 Range 1874 Interquartile Range 220	0.72	-
Hypertension	46 (19.66%)	13 (10.66%)	0.035	1.91(1.06-3.45)
Diabetes Mellitus	31 (13.25%)	1 (0.82%)	<0.001	4.55 (2.12-9.77)
Family History of previous CAD	57 (24.36%)	40 (32.79%)	0.10	0.65 (0.41-1.07)
Waist circumference (cm)	87.44 ± 10.2	83.5 ± 13.72	0.008	-
Metabolic syndrome (IDF criteria)	49 out of 170 (28.82%)	7 out of 121 (5.78%)	<0.001	4.38 (2.43-7.91)
Total cholesterol (mg/dL)	167.92 ± 48.29	151.83 ± 42.27	0.01	-
LDL cholesterol (mg/dL)	100.48 ± 43.23	85.02 ± 29.37	0.003	-
HDL cholesterol (mg/dL)	36.28 ± 9.84	39.54 ± 11.87	0.074	-
Triglycerides (mg/dL)	162.14 ± 93.03	112.71 ± 50.78	<0.001	-
hypercholesterolemia	58 (24.79%)	10 (8.2%)	<0.001	2.92 (1.67-5.09)
Raised LDL	47 (20.09%)	3 (2.46%)	<0.001	4.29 (2.29-8.04)
Reduced HDL cholesterol (<40 mg/dL in males and <50 mg/dL in females)	166 (70.94%)	27 out of 50 (54%)	0.029	2.17 (1.13-4.17)
Increased Triglycerides (≥150 mg/dL)	100 (42.73%)	12 out of 63 (19.05%)	<0.001	2.73 (1.54-4.85)
Central Obesity (IDF criteria)	80 out of 170 (47.06%)	35 out of 121 (28.93%)	0.002	2.19 (1.32-3.43)
Carotid Intimal Medial Thickness (in cm)	0.065 ± 0.002	0.063 ± 0.005	0.761	-
Plaque	8 out of 72 (11.11%)	5 out of 122 (4.1%)	0.076	3.05 (0.95-9.76)
Arcus	32 (13.67%)	13 (10.66%)	0.50	1.31(0.68-2.54)
Premature graying	66 (28.20%)	51 (41.80%)	0.012	0.54 (0.34-0.86)
Baldness	41 (17.52%)	21 (17.21%)	1.00	1.02 (0.57-1.82)

Group 1: Smokers with premature CAD, Group 2: Smokers without premature CAD

The patients in group 1 had increased prevalence of hypertension [19.66% vs 10.66%, OR 1.91 (95% C.I. 1.06-3.45), (*P*=0.035)], diabetes mellitus [13.25% vs 0.82%, OR 4.55 (95% C.I. 2.12-9.77), (*P*<0.001)] and metabolic syndrome [28.82% vs 5.78%, OR 4.38 (95% C.I. 2.43-7.91), (*P*<0.001)] as compared to patients in group 2. There was also increased prevalence of patients with reduced HDL cholesterol [70.94% vs 50.54%, OR 2.17 (95% C.I. 1.13-4.17), (*P*=0.029)], increased triglycerides [42.73% vs 19.05%, OR 2.73 (95% C.I. 1.54-4.85), (*P*<0.001)], central obesity [47.06% vs 28.93%, OR 2.19 (95% C.I. 1.32-3.43), (*P*=0.002)], hypercholesterolemia [24.79% vs 8.2%, OR 2.92 (95% C.I. 1.67-5.09), (*P*<0.001)] and raised LDL [20.09% vs 2.46%, OR 4.29 (95% C.I. 2.29-8.04), (*P*<0.001)].

However, there was no significant difference in presence of family history of CAD [24.36% vs 32.79%, OR 0.65 (95% C.I. 0.47-1.07), (*P*=0.10)], arcus juvenilis [13.67% vs 10.66%, OR 1.31 (95% C.I. 0.68-2.54), (*P*=0.50)] and baldness [17.52% vs 17.21%, OR 1.02 (95% C.I. 0.57-1.82), (*P*=1.00)] in the two groups. Premature graying was found to be more common in group 2 [41.8% vs 28.2%, OR 0.54 (95% C.I. 0.34-0.86), (*P*=0.012)] than group 1. There was also no significant difference in CIMT in 2 groups (0.065 cm vs 0.063cm, *P*=0.761). Plaque was found more commonly in patients in group 1, however, the difference was not statistically significant (*P*=0.076)

## Discussion

In this retrospective case record analysis, we found statistically significant association of hypertension, DM and MS in young smokers with premature acute CAD in Indian population as compared to young smokers without CAD.

CAD has been known to be a disease in which multiple factors like smoking, dyslipidemia, hypertension, diabetes, central obesity and hereditary factors play a major role. Smoking has been shown to be a major dominant modifiable risk factor associated with premature CAD.^9,22,23^ Pais et al. showed that smoking more than 10 bidis in a day resulted in a four times increase in the chances of developing a CAD.^8^ Kannel et al. found in patients included in the Framingham Heart Study, the relative risk for CAD was about three times higher in smokers age 35 to 44, compared to nonsmokers.^24^ This study highlights association of other risk factors including metabolic syndrome, obesity, hypertension and DM with premature CAD independent of the smoking.

Earlier studies have reported prevalence of metabolic syndrome to range from 37% to 60% in patients with premature CAD.^25-27^ We have earlier reported high prevalence (26.8%) of metabolic syndrome in patients with premature CAD.^14^ This study indicates similar prevalence (28.82%) and further highlights significantly higher association of metabolic syndrome in smokers with premature CAD as compared to smokers without CAD. We also report high prevalence of reduced HDL cholesterol, increased triglycerides, LDL cholesterol and total cholesterol in this group of patients and their significantly higher association in smokers with premature CAD as compared to smokers without CAD as shown in Table 1; thus, indicating high unmet need to control lipid profile in patients at-risk for CAD. However, there was no difference in family history of CAD between the 2 groups. Earlier studies have also shown association of corneal arcus and baldness with CAD and premature CAD.^28-31^ Recent studies in Singapore also highlighted association between arcus and CAD after adjusting for smoking.^29,32^ However, our study shows no significant difference in corneal arcus and baldness in smokers with CAD and smokers without CAD. Premature graying of hair has been shown to predispose for coronary artery disease with a study showing that premature graying of hair occurs in patients with CAD under 35 years of age.^33^ Trueb et al suggested that the scalp ageing is subject to intrinsic and extrinsic factors including ultraviolet radiation and smoking.^34^ Surprisingly, we noticed higher prevalence of premature graying in smokers without CAD as compared to smokers with CAD (*P*=0.012). Larger prospective studies may be needed to further clarify the association between these cutaneous markers and premature CAD.

Our study has few limitations. While, it would have been desirable to determine the effect of serum homocysteine levels, lipoprotein (a), small LDL-C, C-reactive protein, psychological factors like stress, diet and lifestyle in the study subjects, it was not logistically feasible for our study group. Since it was a retrospective study, many patient data had to be excluded due to insufficiency of data. Thus, study sample may not be a true representative of the population of premature acute CAD patients at large. Also, we could not get too many controls for more efficient comparison with cases since we choose only patients who presented to out-patient clinics. We acknowledge these limitations. The importance of the study lies in the fact that our study brings out the distinct association of hypertension, central obesity, DM and metabolic syndrome in patients with premature CAD independent of the smoking especially in younger subjects (≤40 years).

## Conclusion

In young smokers, presence of hypertension, central obesity, DM and MS identifies a subset at increased risk for future acute CAD requiring more rigorous follow up and treatment. Intensive monitoring of these patients and strict advocacy of exercise and lifestyle modification are necessary to reduce the prevalence of premature CAD.

## Ethical issues

All patients gave written informed consents and the study was approved by our local Ethics Committee.

## Competing interests

The authors of the present work declare that there is no conflict of interest.
